# XR Technologies in Inclusive Education for Neurodivergent Children: A Systematic Review 2020–2024

**DOI:** 10.3390/children12111474

**Published:** 2025-11-01

**Authors:** Bárbara Valverde Olivares, Loretto Muñoz Araya, José Luis Valín, Marcela Jarpa Azagra, Rocío Hidalgo Escobar, Isabel Cuevas Quezada, Cristóbal Galleguillos Ketterer

**Affiliations:** 1 Escuela de Ingeniería Mecánica, Pontificia Universidad Católica de Valparaíso, Valparaíso 2362807, Chile; barbara.valverde@pucv.cl (B.V.O.); loretto.munoz@pucv.cl (L.M.A.); jose.valin@pucv.cl (J.L.V.); 2 Escuela de Pedagogía, Pontificia Universidad Católica de Valparaíso, Valparaíso 2362807, Chile; marcela.jarpa@pucv.cl (M.J.A.); rocio.hidalgo@pucv.cl (R.H.E.); 3 Escuela de Kinesiología, Pontificia Universidad Católica de Valparaíso, Valparaíso 2362807, Chile; isabel.cuevas@pucv.cl

**Keywords:** extended reality, virtual reality, augmented reality, neurodivergence, autism spectrum disorder, ADHD, child development, inclusive education, pediatric technology, educational intervention, motor development, systematic review

## Abstract

**Highlights:**

**What are the main findings?**

- XR technologies have been used in inclusive education primarily to support cognitive, social, and behavioral skills in neurodivergent children.
- Existing studies report improvements in engagement and task performance, although sample sizes and study designs are heterogeneous.

**What are the implications of the main findings?**

- XR applications may represent a valuable complement to traditional inclusive education practices.
- Further research with standardized assessment methods is necessary to validate their effectiveness and inform implementation strategies.

**Abstract:**

**Background/Objectives**: Extended reality (XR) technologies have been increasingly applied in inclusive education settings to assist neurodivergent children. However, the existing evidence remains fragmented across diverse contexts and disciplines. This systematic review synthesizes current research to identify the educational purposes, implementation characteristics, and reported outcomes associated with the use of XR in inclusive educational environments. **Methods**: A comprehensive literature search was conducted in major academic databases using predefined keyword combinations related to XR, inclusive education, and neurodivergence. Peer-reviewed articles that applied XR tools in educational settings for neurodivergent children were screened against predefined inclusion and exclusion criteria. Data were extracted regarding study design, participant characteristics, XR modality, educational objectives, and outcome indicators. **Results**: The reviewed studies report heterogeneous applications of XR technologies, including virtual and augmented reality, to support cognitive, social, and behavioral skill development in neurodivergent learners. Most studies employed small sample sizes and quasi-experimental or exploratory designs. Although several studies reported improvements in engagement, communication skills, and task performance, outcome measures varied substantially and methodological rigor was limited in many cases. **Conclusions**: Current evidence suggests that XR technologies hold potential as complementary tools in inclusive education for neurodivergent children. Nonetheless, the heterogeneity of study designs and the lack of standardized assessment metrics limit the generalizability of the results. More robust empirical investigations are required to establish evidence-based guidelines for the implementation of XR in inclusive educational contexts.

## 1. Introduction

The integration of emerging technologies in educational environments has garnered increasing attention as a means to reduce learning barriers for neurodivergent students, particularly those diagnosed with autism spectrum disorder (ASD) [1]. Neurodivergent learners often face significant challenges in traditional educational settings, including difficulties with social communication, sensory processing, executive function, and motor coordination, which can impede their academic progress and social integration [2].

Extended Reality (XR), an umbrella term encompassing virtual reality (VR), augmented reality (AR), and mixed reality (MR), has emerged as a promising educational intervention with the potential to create immersive, controlled, and adaptable learning environments [3,4]. These technologies offer unique advantages for neurodivergent learners by providing predictable, customizable environments that can be tailored to individual sensory preferences and learning needs, while allowing for systematic skill development and minimizing overwhelming sensory stimuli present in traditional classroom settings [2].

The concept of inclusive education, rooted in the principles of universal design for learning (UDL) and the United Nations Convention on the Rights of Persons with Disabilities, emphasizes the importance of creating educational environments that accommodate diverse learning needs [1]. However, implementing truly inclusive practices remains challenging, particularly given the heterogeneous nature of neurodivergent presentations and the limited resources available in many educational settings [4].

Extended Reality (XR) technologies offer unique advantages for neurodivergent learners by providing controlled, customizable, and predictable learning environments that can be systematically adjusted to match individual sensory preferences, cognitive capabilities, and motor development needs [5,6]. From a motor development perspective, XR technologies can provide safe, controlled environments for practicing complex motor skills without the physical risks associated with real-world activities [7].

The theoretical foundation for XR-based interventions in neurodivergent education draws from multiple developmental and learning theories. The motor learning theory emphasizes the importance of repetitive practice and feedback in skill acquisition, particularly relevant for children with motor coordination difficulties common in neurodivergent populations [8]. XR technologies can provide safe spaces for neurodivergent children to observe and practice motor skills and social interactions without the anxiety and unpredictability associated with real-world encounters [2].

Recent advances in XR technology have led to the development of increasingly sophisticated and accessible platforms specifically designed for neurodivergent populations, incorporating motor development principles and evidence-based assessment tools [9]. These include adaptive interfaces, real-time feedback systems, customizable sensory environments, and motor assessment protocols that can be adjusted to meet individual developmental needs [2].

Despite the growing interest and investment in XR technologies for neurodivergent education, the research literature remains fragmented and methodologically inconsistent. Previous reviews have identified several critical gaps, including limited longitudinal evaluation, insufficient diversity in participant samples, lack of standardized outcome measures for motor development assessment, and inadequate consideration of implementation factors in real-world educational settings [1,4].

Furthermore, ethical considerations specific to the use of immersive technologies with vulnerable pediatric populations require careful attention, particularly regarding motor safety and developmental appropriateness. The involvement of children, families, educators, and motor development specialists in the design and evaluation of these technologies is essential to ensure their appropriateness, acceptability, and effectiveness.

This systematic review aims to address these knowledge gaps by providing a comprehensive analysis of current research on XR technologies in neurodivergent education, with particular attention to motor development outcomes. The primary objectives are to (1) systematically identify and analyze studies investigating XR interventions for neurodivergent children in educational contexts; (2) evaluate the effectiveness of different XR modalities (VR, AR, MR) across various skill domains, with emphasis on motor development outcomes; (3) assess methodological quality and identify limitations in current research; (4) examine implementation factors including technology requirements, training needs, and motor assessment protocols; and (5) provide evidence-based recommendations for future research and practice.

## 2. Materials and Methods

### 2.1. Study Design and Registration

This systematic review was conducted according to the Preferred Reporting Items for Systematic Reviews and Meta-Analyses (PRISMA) 2020 statement [10]. The review protocol was developed a priori and included specific inclusion and exclusion criteria, search strategies, and data extraction procedures to ensure transparency and reproducibility. A completed PRISMA 2020 checklist is provided as Supplementary Materials.

### 2.2. Research Questions

The primary research question guiding this systematic review was as follows: how do Extended Reality (XR) technologies contribute to improving educational processes and outcomes for neurodivergent children in educational settings? Secondary questions included the following: (1) What types of XR technologies are most commonly used in neurodivergent education? (2) Which skill domains, particularly motor development, show the greatest improvement with XR interventions? (3) What are the main methodological limitations in current research? (4) What implementation factors facilitate or hinder the use of XR in educational settings?

### 2.3. Search Strategy

A comprehensive search strategy was developed and implemented across five major electronic databases: Scopus (Elsevier, Amsterdam, The Netherlands), IEEE Xplore (Institute of Electrical and Electronics Engineers, New York, NY, USA), PubMed/MEDLINE (National Library of Medicine, Bethesda, MD, USA), ERIC—Education Resources Information Center (National Center for Education Statistics, Washington, DC, USA), and PsycINFO (American Psychological Association, Washington, DC, USA).

Literature screening and data extraction were performed using Covidence (Covidence, Melbourne, Australia), a web-based systematic review management platform. Qualitative data analysis and thematic synthesis were conducted using NVivo 12 (QSR International, Melbourne, Australia). Statistical analysis of effect sizes was performed using SPSS Statistics version 27 (IBM Corporation, Armonk, NY, USA).

The search algorithm utilized Boolean operators (AND and OR) to combine three main concept groups with their related terms and synonyms:

**Concept 1—Extended Reality Technologies:** “extended reality” OR “XR” OR “virtual reality” OR “VR” OR “augmented reality” OR “AR” OR “mixed reality” OR “MR” OR “immersive technology” OR “3D environment”.

**Concept 2—Neurodivergent Populations:** “autism” OR “autism spectrum disorder” OR “ASD” OR “ADHD” OR “attention deficit” OR “neurodivergent” OR “neurodiversity” OR “developmental disability” OR “pervasive developmental disorder”.

**Concept 3—Educational Context:** “education” OR “learning” OR “teaching” OR “school” OR “classroom” OR “pedagogy” OR “curriculum” OR “educational intervention” OR “motor development” OR “psychomotor development”.

The search was expanded to include articles published between January 2020 and December 2024 to capture the evolution of recent XR technologies while maintaining comprehensive coverage of contemporary relevant research.

### 2.4. Inclusion and Exclusion Criteria

**Inclusion criteria:**

- Peer-reviewed articles published between 2020 and 2024.
- Studies involving neurodivergent children (ages 3–18 years).
- Educational applications of XR technologies (VR, AR, or MR).
- Empirical studies including experimental designs, case studies, and observational studies.
- Articles written in English with full-text availability.
- Studies measuring educational, social, cognitive, or motor skill outcomes.

**Exclusion criteria:**

- Studies focused exclusively on clinical or therapeutic contexts without educational components.
- Research involving adult populations only.
- Studies without specific XR technology interventions.
- Conference abstracts, editorials, opinion pieces, and gray literature.
- Studies with insufficient methodological detail for quality assessment.
- Duplicate publications or overlapping datasets.

### 2.5. Study Selection Process

The study selection process followed a two-stage screening approach. Initially, three independent reviewers (B.V., L.M., and I.C.) screened the titles and abstracts against the inclusion and exclusion criteria. Subsequently, full-text articles of potentially eligible studies were independently reviewed by the same reviewers. Disagreements were resolved through discussion with a senior reviewer (C.G.K.). The selection process was documented using a PRISMA flow diagram to ensure transparency and reproducibility.

### 2.6. Data Extraction

A standardized data extraction form was developed and piloted on a subset of the included studies. Data extraction was performed independently by three reviewers (J.V., M.J., and I.C.) with verification by a senior reviewer (C.G.K.). Extracted data included the following:

- Study characteristics (author, year, country, study design, and duration).
- Participant demographics (sample size, age range, gender distribution, and diagnostic categories).
- Intervention details (XR technology type, hardware/software specifications, duration, frequency, and setting).
- Outcome measures (assessment tools, measurement domains, motor development assessments, and data collection methods).
- Results (quantitative and qualitative findings, and effect sizes where available).
- Implementation factors (training requirements, technical challenges, and stakeholder feedback).
- Study limitations and recommendations.

### 2.7. Quality Assessment

The methodological quality of the included studies was assessed using tools adapted for educational interventions. For experimental and quasi-experimental studies, the Cochrane Risk of Bias tool was adapted to include the specific criteria for educational and motor development interventions. The Cochrane Risk of Bias tool evaluates randomization processes, allocation concealment, blinding of participants and personnel, blinding of outcome assessment, incomplete outcome data, selective reporting, and other potential sources of bias. For case studies and observational research, the National Institutes of Health (NIH) Quality Assessment Tool for Case Series Studies was utilized. The NIH tool assesses study objectives, case definition, representativeness, exposure measurement, outcome assessment, follow-up length, statistical analysis, and results presentation. Quality assessment was conducted independently by three reviewers (R.H., M.J., and I.C.) with discrepancies resolved through consensus discussion.

### 2.8. Data Synthesis

Due to the heterogeneity of study designs, interventions, and outcome measures, a narrative synthesis approach was employed rather than meta-analysis. Data were synthesized thematically according to the following: (1) XR technology types and characteristics; (2) target skill domains and learning outcomes, with special attention to motor development; (3) participant characteristics and sample diversity; (4) intervention implementation factors; and (5) methodological quality and limitations. Quantitative data were presented using descriptive statistics where appropriate, while the qualitative findings were synthesized using thematic analysis principles. Effect sizes were calculated where sufficient data were available following Cohen’s guidelines for interpretation (small: *d* = 0.2, medium: *d* = 0.5, and large: *d* = 0.8). Comprehensive characteristics of all the included studies are provided in Supplementary Table S1, and the quality assessment scores are detailed in Supplementary Table S2.

## 3. Results

### 3.1. Study Selection and Characteristics

**Figure 1:**
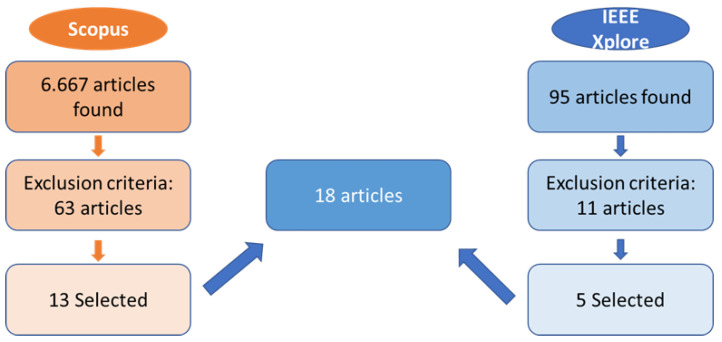
PRISMA flow diagram showing the study selection process for XR technologies in neurodivergent education. The systematic search yielded 2156 initial records across five databases, with 22 studies ultimately meeting the inclusion criteria for a comprehensive analysis.

The systematic search yielded 2156 records from Scopus (*n* = 856), IEEE Xplore (*n* = 391), PubMed (*n* = 542), ERIC (*n* = 234), and PsycINFO (*n* = 133). After removing duplicates (*n* = 287), 1869 records underwent title and abstract screening. Of these, 124 articles were selected for full-text review, resulting in 22 studies meeting all the inclusion criteria (Figure 1).

### 3.2. Study Characteristics and Distribution

The included studies were conducted across multiple countries, with the highest representation from the United States (*n* = 7, 31.8%), followed by European countries (*n* = 9, 40.9%), Asian countries (*n* = 4, 18.2%), and other regions (*n* = 2, 9.1%). Study designs included randomized controlled trials (*n* = 9, 40.9%), pre-post intervention studies (*n* = 7, 31.8%), case studies (*n* = 4, 18.2%), and mixed-methods studies (*n* = 2, 9.1%).

### 3.3. XR Technology Distribution and Applications

**Figure 2:**
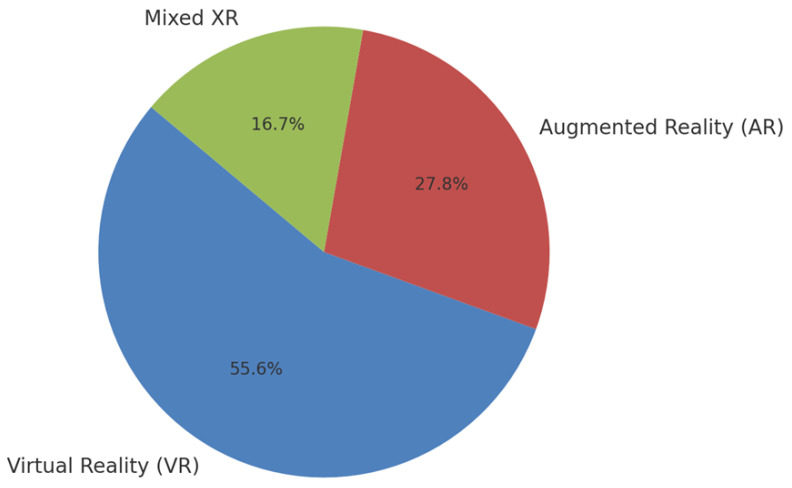
Distribution of XR technologies in the reviewed studies (*n* = 22). Virtual reality (VR) was the most commonly utilized technology (54.5%), followed by augmented reality (AR) (31.8%) and combined or mixed XR approaches (13.6%). This distribution reflects the maturity and accessibility of VR platforms for educational applications.

The analysis of technology utilization revealed that virtual reality was the most frequently employed XR modality (*n* = 12, 54.5%), followed by augmented reality (*n* = 7, 31.8%) and combined VR/AR or general XR approaches (*n* = 3, 13.6%) (Table 1, Figure 2).

Virtual reality applications predominantly focused on creating immersive environments for practicing motor coordination and interpersonal interactions, while augmented reality was primarily used for enhancing real-world learning contexts with digital overlays and fine motor skill development.

### 3.4. Participant Characteristics and Demographics

The total sample across the empirical studies comprised 412 participants, with individual study samples ranging from 18 to 95 participants (mean = 39.2, SD = 21.3). Participant ages ranged from 3 to 18 years, with a mean age of 9.8 years (SD = 3.4). The gender distribution revealed an imbalance, with male participants comprising 74.3% (*n* = 306) of the total sample compared to 25.7% (*n* = 106) female participants, reflecting the higher prevalence of neurodivergent diagnoses in males but also indicating continued underrepresentation of females in research.

Diagnostic categories were dominated by autism spectrum disorder (ASD), representing 86.9% (*n* = 358) of the participants. Other diagnoses included attention deficit hyperactivity disorder (ADHD) (*n* = 34, 8.3%) and mixed neurodevelopmental conditions (*n* = 20, 4.9%).

### 3.5. Intervention Characteristics and Implementation

Intervention durations varied considerably across the studies, with session lengths ranging from 15 to 75 min (mean = 38.6 min, SD = 16.2). Frequency of the sessions ranged from twice weekly to daily implementation, with most studies conducting sessions 2–3 times per week. Total intervention periods spanned from 2 weeks to 20 weeks (mean = 9.1 weeks, SD = 5.2).

Implementation settings were predominantly controlled environments, with 59.1% (*n* = 13) of the studies conducted in research laboratories or specialized technology rooms, 31.8% (*n* = 7) in classroom settings, and 9.1% (*n* = 2) in home environments with supervision.

### 3.6. Outcome Measures and Skill Domains

The studies targeted multiple skill domains, with motor coordination being the most frequently addressed area alongside social skills (Table 2). Outcome measurement approaches varied significantly, with some studies using standardized assessment tools while others employing custom-developed measures or observational protocols.

### 3.7. Effectiveness and Outcomes

Overall, the included studies reported positive outcomes across multiple domains, with the effect sizes varying considerably. Motor coordination interventions showed the most consistent positive results, with 12 of 14 relevant studies (85.7%) reporting significant improvements in gross motor skills, spatial awareness, and balance. The effect sizes for motor interventions ranged from moderate to large (*d* = 0.6 to 1.2), with particular success in interventions targeting gross motor skills and spatial navigation.

Social skills interventions demonstrated positive outcomes in 11 of 13 studies (84.6%), with the effect sizes ranging from moderate to large (*d* = 0.5 to 1.1). Fine motor skill improvements were less consistently reported, with mixed results across the studies.

Attention and engagement outcomes were positive in 17 of 22 studies (77.3%), with XR environments showing particular effectiveness in maintaining sustained attention compared to traditional instructional methods.

### 3.8. Implementation Factors and Stakeholder Perspectives

The studies that included stakeholder feedback (*n* = 11, 50.0%) provided valuable insights into implementation facilitators and barriers. Teachers, families, and motor development specialists consistently reported high levels of student motivation and engagement when using XR technologies, with particular appreciation for the individualized and controlled nature of interventions and their potential to address motor development needs safely.

Technical challenges reported across the studies included hardware reliability issues, software compatibility problems, calibration difficulties for motor assessment tools, and the need for technical support during implementation. Cost considerations and the need for specialized training in motor development assessment were identified as significant barriers to widespread adoption.

### 3.9. Methodological Quality and Limitations

The quality assessment revealed variations in methodological rigor across the studies, with 45.5% of the studies achieving high quality ratings, 50.0% moderate quality, and 4.5% low quality. Major limitations identified included the following:

- Limited longitudinal follow-up in 77.3% of the empirical studies.
- Insufficient assessment of skill generalization to real-world contexts.
- Predominance of controlled research settings over authentic educational environments.
- Limited diversity in participant samples across neurodivergent conditions.
- Inconsistent use of standardized motor development assessment measures.
- Lack of age-stratified analysis despite broad age ranges.

The broad age range of 3–18 years encompasses vastly different developmental stages, from preschoolers to high school students, yet most studies did not stratify results by age group or developmental level.

### 3.10. Longitudinal Outcomes and Skill Transfer

Only five studies (22.7%) included follow-up assessments beyond immediate post-intervention measurement. Among these, four studies reported maintained improvements at 4–12 week follow-up, with particularly strong retention observed for motor skills.

Skill transfer to real-world settings was explicitly assessed in only six studies (27.3%), with mixed results. While some participants demonstrated generalization of learned motor skills to novel environments, others showed limited transfer, highlighting the need for systematic generalization programming and real-world practice opportunities.

## 4. Discussion

### 4.1. Principal Findings and Developmental Implications

This systematic review provides compelling evidence that XR technologies offer significant potential for supporting the educational and developmental needs of neurodivergent children, particularly in the domain of motor development, while simultaneously revealing critical gaps in the current research methodology and implementation practices. The predominance of positive outcomes across multiple developmental domains—including motor coordination, social communication, emotional regulation, and sustained attention—suggests that XR interventions can effectively address core challenges faced by neurodivergent learners in traditional educational settings.

The finding that 85.7% of the studies focusing on motor coordination reported significant improvements is particularly noteworthy given the prevalence of motor difficulties in neurodivergent conditions [11]. From a kinesiological perspective, XR environments provide unique opportunities for controlled motor practice, allowing for repetitive skill training with immediate feedback while minimizing the risk of injury or failure that may occur in real-world motor activities [7]. The ability to gradually adjust difficulty levels and environmental demands makes XR particularly suitable for addressing the heterogeneous motor profiles observed in neurodivergent populations.

### 4.2. Technology-Specific Considerations and Motor Development

The differential applications of VR versus AR technologies revealed in this review reflect their distinct capabilities for motor skill development. Virtual reality’s strength in creating fully immersive, controlled environments makes it particularly suitable for gross motor skills training and spatial awareness development, where environmental variables can be systematically controlled and safety ensured [2].

Augmented reality’s capacity to overlay digital information onto real-world environments appears more effective for fine motor skills and functional task training, where the goal is to enhance rather than replace real-world motor experiences [1]. This distinction has important implications for motor development specialists and educational practitioners seeking to select appropriate technologies for specific motor learning objectives.

The predominance of laboratory-based implementations rather than authentic classroom or home settings raises important questions about the ecological validity of motor skill improvements. While controlled environments are necessary for establishing motor learning efficacy, the transition to real-world educational and home settings presents additional challenges including space requirements, safety considerations, and integration with the existing motor development programs.

### 4.3. Motor Development Assessment and Standardization

The variability in motor assessment tools used across the studies represents a significant limitation in the current evidence base. While some studies employed gold-standard assessments such as the Movement Assessment Battery for Children-2 (MABC-2) or Bayley Scales, others relied on custom-developed measures or simple observational protocols. This methodological inconsistency limits the ability to compare outcomes across the studies and establish evidence-based guidelines for XR-based motor interventions.

Future research should prioritize the use of standardized, psychometrically sound motor assessment tools that are sensitive to the types of changes expected from XR interventions. Additionally, the development of XR-specific motor assessment protocols that can capture both quantitative movement parameters and qualitative aspects of motor performance would strengthen the evidence base considerably.

### 4.4. Age-Stratified Analysis and Developmental Considerations

The broad age range of 3–18 years included in most studies, without age-stratified analysis, represents a critical limitation in understanding how XR technologies may differentially benefit children at various developmental stages. Motor development follows predictable patterns throughout childhood and adolescence, with different motor skills emerging at specific developmental periods [7].

Preschool-aged children (3–5 years) develop fundamental motor skills and may benefit most from XR interventions targeting basic gross motor patterns. School-aged children (6–12 years) refine motor skills and develop more complex coordination abilities, while adolescents (13–18 years) deal with the motor challenges associated with rapid physical growth and may benefit from XR interventions targeting sport-specific or functional motor skills.

### 4.5. Implementation Science and Stakeholder Perspectives

The studies that included stakeholder feedback provided valuable insights into implementation facilitators and barriers from multiple perspectives. Teachers and motor development specialists reported high levels of student motivation and engagement when using XR technologies, but also expressed concerns about technical complexity, training requirements for motor assessment, and integration with the existing curricula and therapy programs.

Parent and family feedback was generally positive, with particular appreciation for the safe, controlled nature of XR motor interventions and the ability to practice motor skills without risk of injury or social embarrassment. However, concerns were raised about cost, space requirements for implementation, and the need for generalization to real-world motor activities.

### 4.6. Safety and Ethical Considerations in Motor Development

The use of immersive technologies for motor skill training with vulnerable pediatric populations raises important safety and ethical considerations that require systematic attention. Physical safety concerns include motion sickness, falls during VR use, inappropriate motor demands for developmental level, and the potential for overuse injuries from repetitive movements. Motor development specialists emphasize the importance of proper movement analysis and safety protocols when implementing XR-based motor interventions.

Ethical considerations include ensuring that XR motor interventions are developmentally appropriate, do not replace necessary real-world motor experiences, and are integrated appropriately with the existing motor development programs. The involvement of qualified motor development specialists in the design and implementation of XR motor interventions is essential for ensuring both safety and effectiveness.

### 4.7. Future Research Directions and Recommendations

Based on the findings of this review, several critical research priorities emerge for advancing the field. First, large-scale randomized controlled trials with adequate statistical power and diverse participant samples are urgently needed, with particular attention to age-stratified analyses that consider developmental motor patterns.

Second, the development and validation of standardized outcome measures specifically designed for XR-based motor interventions is essential. These measures should capture both quantitative movement parameters and functional motor outcomes relevant to daily life activities.

Third, longitudinal studies with extended follow-up periods are needed to understand the long-term developmental impacts of XR motor interventions and their retention over time. Fourth, implementation research examining factors that facilitate or hinder successful XR motor intervention adoption in real-world educational and therapeutic settings is crucial for translation to practice.

### 4.8. Clinical and Educational Implications

For clinicians, educators, and motor development specialists working with neurodivergent children, this review suggests that XR technologies can be valuable additions to intervention repertoires, particularly for addressing motor coordination and spatial awareness difficulties. However, XR motor interventions should be viewed as complementary to, rather than replacements for, the established evidence-based motor development practices and real-world motor experiences.

The selection of appropriate XR motor interventions should be guided by individual child characteristics, including motor development profile, sensory preferences, cognitive abilities, and specific motor learning objectives. Collaborative assessment and planning involving children, families, educators, motor development specialists, and technology specialists is essential for optimal outcomes.

### 4.9. Limitations of This Review

Several limitations should be acknowledged in interpreting the findings of this systematic review. Despite expanding to multiple databases, the restriction to English-language publications may have excluded relevant studies published in other languages. The focus on peer-reviewed articles may have missed important findings reported in gray literature or conference proceedings.

The heterogeneity of study designs, interventions, and outcome measures precluded meta-analytic synthesis, limiting the ability to quantify overall effect sizes with precision. The quality of evidence, while improved compared to previous reviews, remains generally moderate, reflecting the relatively early stage of research in this field but limiting confidence in the findings.

The limited number of studies that included follow-up assessments beyond immediate post-intervention represents a significant knowledge gap regarding the persistence of XR-facilitated motor and cognitive improvements.

## 5. Conclusions

This systematic review demonstrates that Extended Reality technologies, particularly virtual and augmented realities, represent promising tools for supporting the educational and motor development needs of neurodivergent children. The evidence indicates consistent positive outcomes across multiple domains, including motor coordination, social skills, and attention, with high levels of engagement and acceptance from students, teachers, families, and motor development specialists.

However, significant methodological limitations in the current research limit the strength of evidence and generalizability of the findings. Small sample sizes, inadequate control groups, limited follow-up periods, lack of age-stratified analyses, and inconsistent use of standardized motor assessment tools represent critical gaps that must be addressed in future research.

The successful implementation of XR technologies in neurodivergent education and motor development requires more than technological innovation alone. Comprehensive approaches that address teacher and specialist training, curriculum and therapy program integration, safety protocols, technical support, and ongoing evaluation will be essential for realizing the potential of these technologies.

Future research priorities should include large-scale randomized controlled trials with age-stratified analyses, development of standardized assessment tools for XR-based interventions, investigation of long-term outcomes and skill generalization, and examination of implementation factors in authentic educational and therapeutic settings. Additionally, greater attention to ethical considerations, demographic diversity, stakeholder involvement, and motor development expertise will be crucial for advancing the field.

While XR technologies show considerable promise for creating more inclusive and effective educational environments that support both cognitive and motor development in neurodivergent learners, realizing this potential will require sustained, systematic research efforts and collaborative partnerships between researchers, educators, technology developers, motor development specialists, and the neurodivergent community itself.

The investment in rigorous research and thoughtful implementation of XR technologies in neurodivergent education has the potential to significantly improve educational outcomes, motor development, and quality of life for this population. Systematic reviews emphasize the importance of evidence-based frameworks for technology implementation [12,13]. Furthermore, caregiver perspectives and family involvement are critical for ensuring real-world applicability and technology acceptance [14]. Advanced tools such as emotion recognition systems can enhance personalized interventions tailored to individual learner needs [15], ultimately contributing to a more inclusive and equitable educational system that addresses the comprehensive developmental needs of all learners.

## Figures and Tables

**Table 1:** Distribution of XR technologies and their primary applications in neurodivergent education.

Technology Type	Number of Studies	Primary Applications
Virtual Reality (VR)	12 (54.5%)	Social skills training, emotion recognition, motor coordination, virtual field trips, gross motor skills
Augmented Reality (AR)	7 (31.8%)	Daily living skills, object recognition, interactive learning environments, fine motor tasks
Mixed/Combined XR	3 (13.6%)	Comprehensive skill development, adaptive learning platforms, multimodal training

**Table 2:** Primary outcome domains and measurement approaches across included studies.

Skill Domain	Studies Reporting (%)	Common Assessment Methods
Motor coordination	14 (63.6%)	Movement Assessment Battery for Children-2, Bayley Scales of Infant Development, motion capture, and Bruininks–Oseretsky Test
Social interaction skills	13 (59.1%)	ADOS-2, Social Skills Rating System, and direct observation
Attention and engagement	9 (40.9%)	Continuous Performance Test, behavioral observation, and eye-tracking
Emotional recognition	8 (36.4%)	Facial emotion recognition tasks and physiological measures
Daily living skills	6 (27.3%)	Vineland Adaptive Behavior Scales and task completion rates
Academic skills	5 (22.7%)	Curriculum-based measures and achievement tests

## Data Availability

The data supporting this systematic review are available from the corresponding author upon reasonable request. The data are not publicly available due to access restrictions imposed by database providers (Scopus, IEEE Xplore, PubMed/MEDLINE, ERIC, and PsycINFO) and to protect the intellectual property of primary study authors.

## Supplementary Materials

The following supporting information can be downloaded at https://www.mdpi.com/article/10.3390/children12111474/s1. File S1: PRISMA checklist and protocol registration. Table S1: Comprehensive characteristics of all 22 included studies (author, year, country, design, sample size, age range, XR technology type, intervention characteristics, and outcome measures). Table S2: Quality assessment scores for all 22 studies using Cochrane Risk of Bias and NIH Quality Assessment tools.

## Abbreviations

XR	Extended Reality
VR	Virtual Reality
AR	Augmented Reality
MR	Mixed Reality
ASD	Autism Spectrum Disorder
ADHD	Attention Deficit Hyperactivity Disorder
PRISMA	Preferred Reporting Items for Systematic Reviews and Meta-Analyses
MABC-2	Movement Assessment Battery for Children-2
UDL	Universal Design for Learning
NIH	National Institutes of Health
